# Manhattan Harvester and Cropper: a system for GWAS peak detection

**DOI:** 10.1186/s12859-019-2600-4

**Published:** 2019-01-11

**Authors:** Toomas Haller, Tõnis Tasa, Andres Metspalu

**Affiliations:** 1 Estonian Genome Center, Institute of Genomics, University of Tartu, 23b Riia Street, 51010 Tartu, Estonia; 2 Institute of Computer Science, University of Tartu, Juhan Liivi 2, 50409 Tartu, Estonia

**Keywords:** GWAS, Manhattan plots, Peak detection, Peak quality score, Software

## Abstract

**Background:**

Selection of interesting regions from genome wide association studies (GWAS) is typically performed by eyeballing of Manhattan Plots. This is no longer possible with thousands of different phenotypes. There is a need for tools that can automatically detect genomic regions that correspond to what the experienced researcher perceives as peaks worthwhile of further study.

**Results:**

We developed Manhattan Harvester, a tool designed for “peak extraction” from GWAS summary files and computation of parameters characterizing various aspects of individual peaks. We present the algorithms used and a model for creating a general quality score that evaluates peaks similarly to that of a human researcher. Our tool Cropper utilizes a graphical interface for inspecting, cropping and subsetting Manhattan Plot regions. Cropper is used to validate and visualize the regions detected by Manhattan Harvester.

**Conclusions:**

We conclude that our tools fill the current void in automatically screening large number of GWAS output files in batch mode. The interesting regions are detected and quantified by various parameters by Manhattan Harvester. Cropper offers graphical tools for in-depth inspection of the regions. The tools are open source and freely available.

**Electronic supplementary material:**

The online version of this article (10.1186/s12859-019-2600-4) contains supplementary material, which is available to authorized users.

## Background

For over a decade the genome-wide association studies (GWAS) have been a powerful tool in the arsenal used for unraveling the information present in the genome [1]. Despite certain skepticism this approach is not showing signs of fatigue. Quite to the contrary, the number of GWAS carried out is increasing, returning useful information for understanding the genome and predicting and helping to cure disease [2]. All this paves the road for personalized medicine – bound to become the backbone of the medicine in the future. With the increasing number of genotyped and sequenced individuals as well as advances in high performance computing the GWAS projects undertaken have grown in size and technological complexity [3]. There are reports out that have boosted the number of individual phenotypes in some cases to tens of thousands or more [4]. It is not rare to combinatorially generate even more phenotypes (e.g. metabolite ratio phenotypes) and analyze in one go [5]. These results can no longer be individually evaluated by a researcher. Automatic screening of results is much needed for a quick summary of the findings and to rank them in the order of significance. Yet well documented specific tools for this purpose are still missing to the best of our knowledge. We present Manhattan Harvester (MH) that uses the GWAS output files and detects the signals (peaks) of potential interest from them by mimicking the eye of a researcher. The software computes a list of parameters for each peak and a quality score based on these. MH is supplemented by another original tool – Cropper. Cropper is a visual aid for viewing, zooming, cropping and subsetting GWAS results. It can be used in combination with MH when studying the findings of MH.

## Implementation

### Scripting and properties

Both MH and Cropper are written in C++/Qt [6]. They are open source and can be downloaded from www.geenivaramu.ee/en/tools. It is possible to compile them for all major computational platforms. Both tools are fully documented and accompanied by instructions and examples.

### Manhattan harvester (MH)

**Fig. 1:**
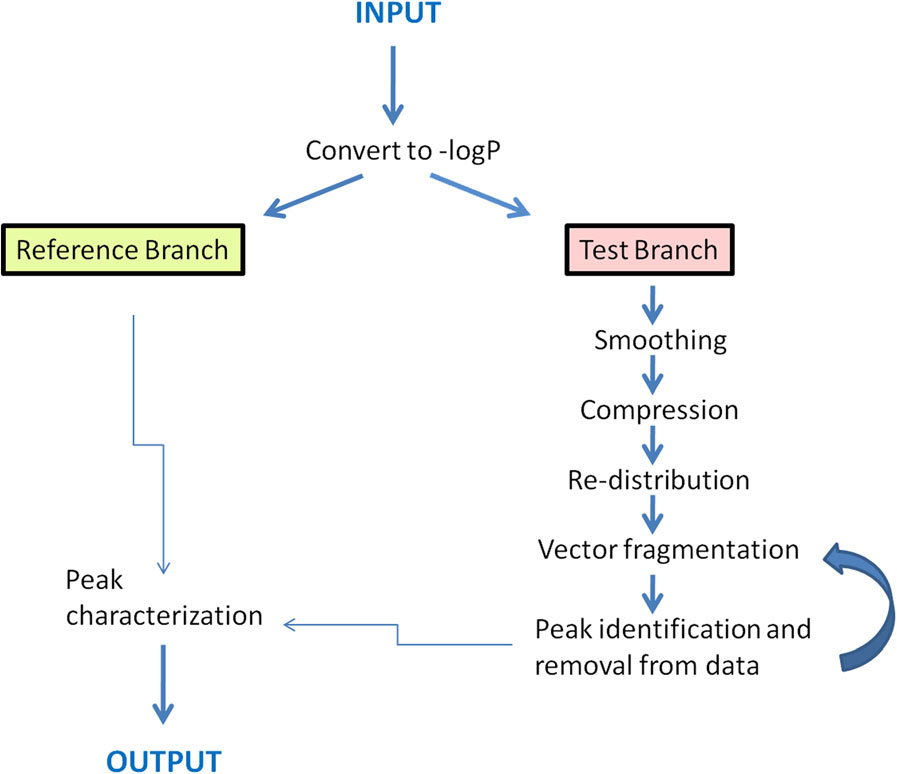
Workflow of MH

**Fig. 2:**
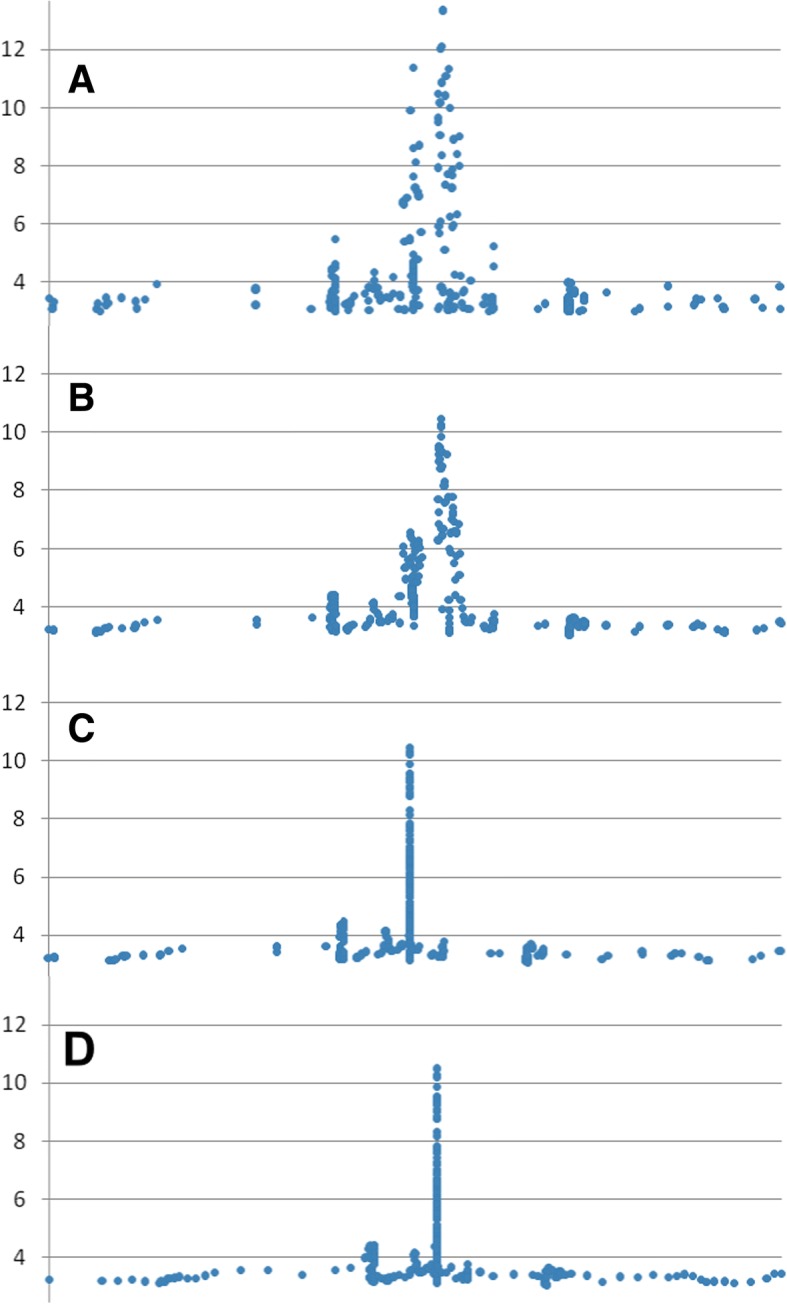
The key steps of data processing in the Test Branch of MH. **a**: original (raw) data, **b**: smoothing, **c**: height-based compression, **d**: local range re-distribution. The Y axis is -log(*p*-value), the X axis is physical position. The absolute position values can be different between different panels of the graph, they were scaled based on the first and last data point position

**Fig. 3:**
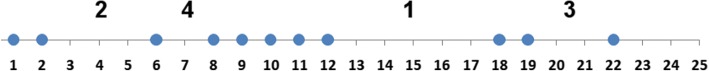
The order of chunk creation during vector fragmentation by MH. The numbers indicate the order of gaps by size. The first fragmentation round (1) yields 8 points, (2) yields 6 points, (3) is not executed because the corresponding area was lost after step (1), and (4) yields 5 points – the densest area of the plot

MH is a command line tool working on GWAS output files. It is able to analyze all chromosomes together or one at a time and can operate in single file or batch mode. MH provides the user with a table containing all physical position regions (peaks) detected in the GWAS output, the peak parameters and their general quality scores (see below). It utilizes original and efficient algorithms to handle the GWAS files. MH starts by reading rows with valid position numbers and *p*-values under a certain threshold (p-value< 0.001 as the default). Two copies of the data sets are handled in parallel – one remains unchanged (Reference Branch), the other one (Test Branch) is modified by various functions required for signal detection. Later the information from the two branches is merged to get the final annotations (Fig. 1). The modifications performed in the test branch standardize the input so that the peaks (nearby data points representing local regions of low *p*-values) can be separated from the background noise. The test branch data undergo the following modifications:

- Signal smoothing. The data are smoothed using a sliding window. Linear regression is performed sequentially for each data point by making use of 5 data points (2 before and 2 after). The middle data point is replaced with its prediction from linear regression (Fig. 2, a-b).
- Height-based compression. The spaces between data points are compressed based on the average -log(*p*-value) of their flanking data points multiplied by a constant (default value = 2). This step ensures that the points with small *p*-values (and hence more likely to belong to the peaks) are compressed closer to one another than the points corresponding to the intermediate space between the peaks (Fig. 2, c). As an example to illustrate this step: points A (-logP_A_ = 4) and B (-logP_B_ = 10) that are originally 1000 bp apart become 100 bp apart ($$ \raisebox{1ex}{$1000\  bp$}\!\left/ \!\raisebox{-1ex}{$\left(2\ast \left(4+6\right)/2\right)$}\right. $$).
- Local-range re-distribution. From this step forward the *p*-values of the points are ignored as the algorithm continues only with a one dimensional projection of the compressed (see step b) physical position values. In this step all points are evenly distributed between their neighboring data points. This is done in two stages so that each point is slid along the position axis relative to two secure anchor points. This means that every other point is relocated using its neighbors as anchors, then the anchors themselves are relocated using their own flanking points as anchors. For example consider sequential points 1, 2, 3, 4, 5 that have variable distances between them. In the first stage point 2 is set to equal distance from 1 and 3 and point 4 is set to equal distance from 3 and 5. In the second stage point 3 is set to equal distance from 2 and 3. This re-distribution ensures that the distances between points are more evenly distributed - a prerequisite for the next step. The points in the regions falling between the peaks relocate much more than those in the peak regions because the latter are locked tight between their neighbors and they have less space to relocate (Fig. 2, d). The order of points is never altered. As a result the difference between the largest gap found inside the peaks and the smallest gap found in the inter-peak region is widened; essentially the peak points become more distinguishable from the background. This is relevant because the peak regions are now differentiated from the inter-peak regions only by the data point density in the one-dimensional array.
- Vector fragmentation. We modified the framework of univariate clustering [7] for our specific needs. Our vector fragmentation procedure is searching for the optimal clusters within the physical position values space of the chromosome. It is a carried out on the standardized input (step c) and the outcome is the genome regions that constitute Manhattan Plot peaks. These regions are separated from the flanking regions by sequential fragmentation of the position values array. The chunks are created by iteratively breaking the vector where the distances between the points are the largest, gradually moving to the smallest. Always the chunk with more data points is carried over to the next round of fragmentation (Fig. 3). During such fragmentation there is a termination point that optimally corresponds to the peak with the densest point distribution. To pinpoint the best stopping point the mean inter-point gap size (*meanG*) and the maximal inter-point gap size (*maxG*) are recorded for each fragmentation step. Two parameters are computed for each chunk: a) $stop{1}_i=\frac{\mathit{\max}{G}_i}{mean{G}_i}$, b) $stop{2}_i=\frac{\mathit{\max}{G}_i}{\mathit{\max}{G}_{i+1}}$, where *i* is the fragmentation step index. The optimal chunk was found to correspond to the index *i* of $\underset{\mathrm{i}\in \mathrm{n}}{\max } stop{1}_i$, or else $\underset{\mathrm{i}\in \mathrm{n}}{\max } stop{2}_i$ if *stop*1_*i*_ − *stop*2_*i*_ > 2. This empirical solution to choose the best fragmentation stopping point eliminated the need for more complicated decision making structures and proved fully adequate for analyzing real data. Larger stop1 and stop2 values generally correspond to the inter-peak regions whereas small values are indicative of fragmentation cuts in the middle of the peaks. Hence the borders where these values turn from large to small align with the peak borders. In addition to this detection system MH also applies several “sanity check” filters such as the maximal height to width ratio, chunk size etc. to narrow down the options space for the stop1/stop2 fragmentation termination system. The last filter in the algorithm is a function that tests the left and right *p*-values of the newly detected peak candidates to decide whether the next smallest chunk size has more fitting left and right peak termini in terms of p-value (as decided relative to the smallest peak height and baseline p-values); in which case the next smallest chunk is selected instead. MH comes optimized with regard to the analytical parameters as the default values. However, all key parameters can be changed by the user via command line flags as the need arises (see MH manual).
- Peak characterization and re-looping. Once the peak borders are identified the peak is characterized by a number of parameters. This includes for example General Quality Score (GQS, see below), maximal slope, height to width ratio and more (see Table 1 of Additional file 1). These parameters can be used to let the user filter and prioritize the findings. After this step the data points corresponding to the peak are removed from the data and the algorithm loops back to step d for the next round of vector fragmentation and the identification of the peak with the second highest point density (Fig. 1). The cycle between vector fragmentation, characterization of the created fragments and removal of the characterized fragments continues until data points are depleted.

**Table 1:** Execution speed of MH with various input file sizes, number of detected peaks and computational systems

Min p-val	Peaks	File size (MB)	Data points (rows)	PC, sec (mean ± stdev)	HPC, sec (mean ± stdev)
0.01	1	0.257	8507	0.037 ± 0.0018	0.037 ± 0.0045
0.01	3	0.348	11,781	0.052 ± 0.0049	0.056 ± 0.011
0.01	5	0.215	7116	0.031 ± 0.0018	0.033 ± 0.0033
< 1	6	85	560,646	NA	3.07 ± 0.0758

### Cropper

**Fig. 4:**
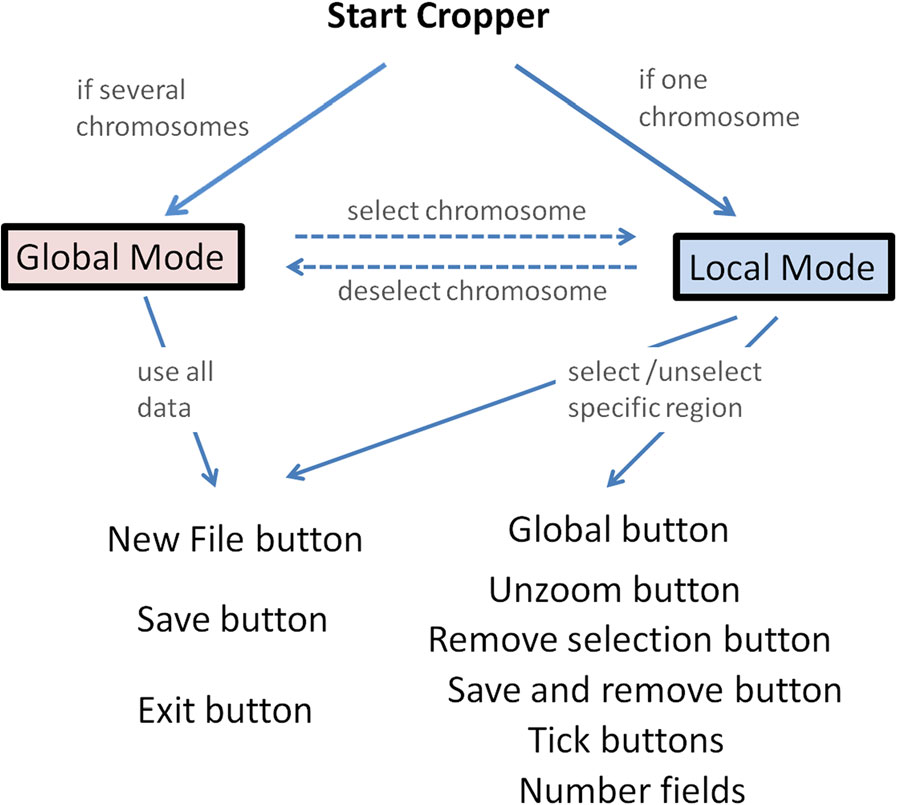
A summary of how Cropper works. The two views (global and local) make different control options available to the user

Cropper is a GUI tool using standard data visualization logic and patterns. It is specifically designed for handling Manhattan Plots. Cropper was developed in synchronization with the demands that originated during MH production, validation and usage. The user can zoom, crop and output parts of Manhattan Plot in both graphical and numerical format. Cropper also allows to sequentially remove peaks from Manhattan plot so that the user can continue work with the leftover data set after cropping out peaks. Cropper offers two views: a) global view showing all chromosomes, b) local view showing the selected chromosome (Fig. 4). Chromosomes are chosen from the global view while all the selections and manipulations are done in the local view by using the mouse (see Fig. 1 in Additional file 1). It is easy to visualize the regions picked out by MH by copying their ranges directly from the MH output file to the range data field of Cropper.

## Results

### Data

In this work we used the NMR metabolite GWAS meta-analysis data set from the MAGNETIC consortium which is freely available [8, 9]. The files had a GWAMA format [10]. The data files were randomly divided into two non-overlapping subsets: method development set (MDS) and the method validation set (MVS).

### General quality score (GQS)

**Fig. 5:**
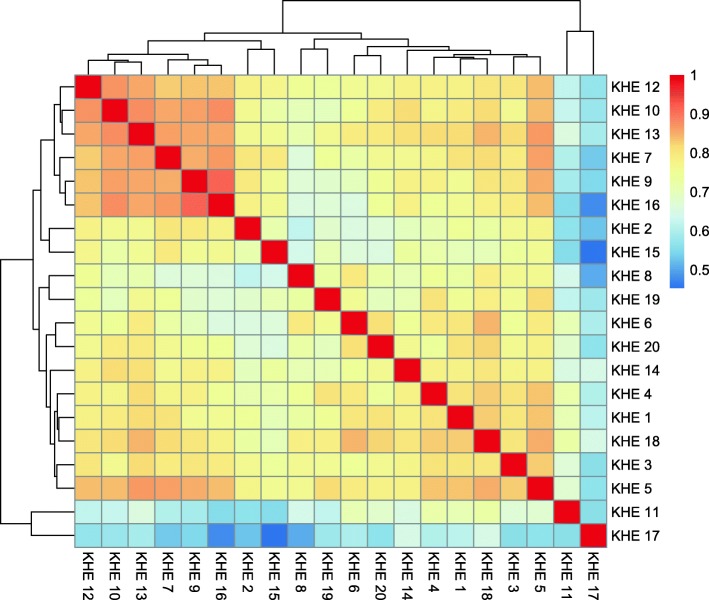
A heat-map with hierarchical clustering showing the Manhattan Plot peak assignment groupings between KHEs

MH computes 16 parameters for each peak. Each parameter describes a certain aspect of the peak region and can be used for subjective ranking (see Table 1 in Additional file 1). We built a model to predict the “goodness” of GWAS peak based on these values to generate a GQS for each peak. The more comprehensive GQS score was invented to provide a more global quality assignment for each peak that could be used as the main parameter for peak assessment. The peak quality score model was created using the quality scores assigned by the volunteer knowledgeable human evaluators (KHEs, the scientists from the Institute of Genomics, University of Tartu, knowledgeable in GWAS) as dependent variables. We collected the benchmark data set by asking 20 KHEs to evaluate 277 Manhattan Plot peaks (extracted with Cropper from the MDS) on a 5 point scale. These peaks were shown to the KHEs together with flanking areas (1/3 before and 1/3 after the peak region). Each KHE could also see the maximal –log(*p*-value) for the top of the peak and the width of the peak at p-value = 0.01. The evaluation marks generally agreed well between the KHEs as witnessed by the grading correlation structure (Fig. 5). The correlation structure within the set of parameters used for modeling was investigated (see Fig. 2 in Additional file 1). Many parameters showed either a strong positive or negative correlation with the marks given by the KHEs and in most cases the variation was low (see Fig. 3 and Fig. 4 in Additional file 1).

**Fig. 6:**
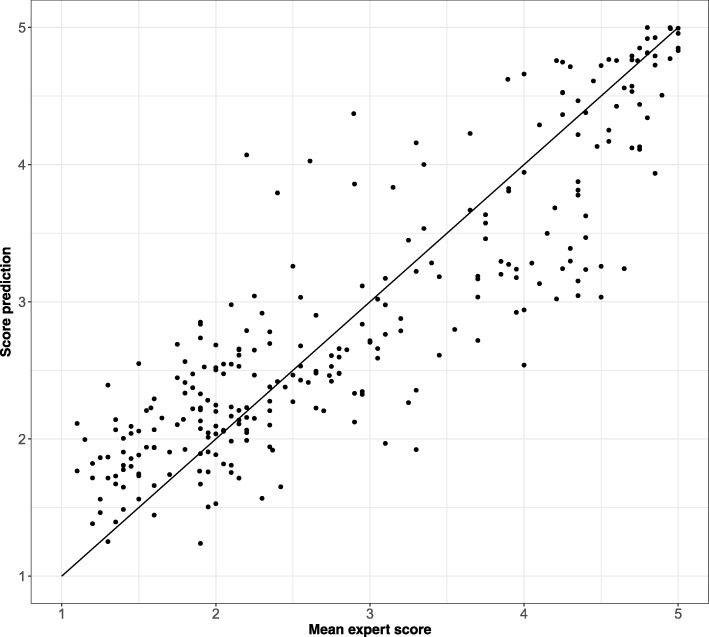
Validation of the statistical model to estimate GQS – expected scores (based on KHE marks) vs. observed (computed by MH)

We used the KHE-generated peak scores together with the16 parameters generated by MH as attributes to model the outcome grade variables with a mixed-effects proportional odds model [11]. The attribute “peak repetition” was transformed by taking the square-root, the attributes “skewness” and “peak balance” were raised to the power of 0.25 and all other attributes except “Kolmogorov normality test” were log-transformed (see Table 1 in Additional file 1). All variables were additionally mean scaled and standardized. The model used was a mixed-effects proportional odds model with a cumulative link that assumes an ordinal response of the peak scores. These scores are assumed to be subject to expert specific effects. We used a step-wise model development approach starting from the null model that minimized the average mean square error ($1/\mathrm{n}{\sum}_{i=1}^n{\left(\underset{i}{\Pr }-T{r}_i\right)}^2$ where *Pr*_*i*_ is the *predicted grade* of the i_th_ graded peak, *Tr*_*i*_ is *true grade* given to a peak and n is the number of graded instances of the predictions using five-fold out-of-sample cross validation. The initial dataset was split five-fold and the model was trained five times using 4 of the 5 folds and using the fold left-out fold as test data. We used R (version 3.3.3) package ordinal (version 2018.4–19) for model development [12]. Of all parameters “log max *p*-value” and “bestslope” were incorporated into the final model. This was the optimal solution that resulted in minimum 5-fold cross validation MSE of 0.92. The final model parameters were obtained from refitting the final model on all data. This resulted in Pearson correlation coefficient of *r* = 0.88 between the expected value of predicted scores (E (score)=$\sum \limits_{i=1}^5P\left( score=i\right)\ast i$) and the mean estimate from 20 KHE GQSs (Fig. 6). The R code used for modeling is presented in Additional file 2. The model was implemented in MH.

### Manhattan harvester peak detection accuracy

It is important to evaluate the implemented algorithms of MH on test data to show how well they detect the Manhattan Plot peak regions and estimate peak quality. The adequacy of the MH algorithm can be evaluated relative to the KHE estimations. It is not preferable to use known (published) biological significance of the detected regions as a quality measure because the quality of the peak is not linked to biological significance. Instead it is the peak height, width and shape that typically mark a peak as interesting for the next steps of the study. We assessed the MH output by comparing the MH-detected regions with the opinion of a KHE using the MVS. Cropper was used by the KHE to conduct the comparison between the Manhattan Plot and the regions picked out as peaks by MH. The KHE inspected 100 randomly chosen peaks ranging from 5.1 to 81.7 on a –log(*p*-value) scale. Of those 97 coincided very closely with the KHE opinion, only one peak (max(−log(p-value)) = 39.1) was detected as to have a base range two times wider (extended equally to the left and right of the highest point) than marked by the KHE. Massive peaks were fragmented into 2 or 3 sub-peaks on two occasions (separating the center of the peak region from the shoulders). It was not unambiguous, however, if this solution was better or worse than reporting those peaks (3 MB wide at the height of –log(p-value) = 3) as single clumps. In no cases were the existing peaks not reported and the reported peak ranges never overlapped as judged by the KHE. A KHE also visually validated the GQS on the data used to validate the results above. The GQS values computed by MH were always within one unit of the score visually assigned by the KHE using Cropper.

### Computational speed

The computational speed of MH was measured by analyzing MDS files on two systems: a) Hewlett Packard EliteBook 2540p (PC), with 14.04.5 LTS (32 bit), 2 GHz Processor, b) High Performance Computing (HPC) cluster of the University of Tartu running CentOS Linux release 7.4 (64 bit) and 2.2 GHz processor. All measurements were carried out 5 times on a single processor using the Unix command “time”. We used minimal (filtered) file size with *p*-val < 0.01 (< 1 MB) as well as an un-modified GWAMA meta-analysis file (85 MB) (Table 1).

We show that an 85 MB GWAMA format file with 19 columns, 560,646 rows and 6 genomic regions detected by MH was analyzed in just over 3 s (see Fig. 5 in Additional file 1 for Manhattan Plot). The analysis took 0.031–0.056 s when file reading time was minimized. We did not detect association between execution speed and the number of peaks detected. Most of the computational time was spent on file reading and the overall analysis speed was sufficiently fast for practical purposes.

The execution speed of Cropper was not quantified. It was qualitatively judged by the users (KHEs) as sufficiently fast.

### Cropper evaluation

Cropper was initially created to facilitate the development process of MH. It gained a role as a tandem tool in the MH workflow – used for visual validation of MH assignments. The other graphical tools that could be adopted for viewing or cropping of Manhattan Plots were not convenient for a quick visual assessment of the findings from MH. Cropper proved uniquely valuable for quickly conducting the comparison between the peak assignments by MH and KHE for this project both in terms of ease of use and speed. The capability of Cropper to visualize regions from MH output file in one copy and paste step proved most useful. It was specifically brought out by the KHEs that Cropper had no easy-to-use alternatives for fast visualization of selected Manhattan plot regions.

## Discussion

To this day GWAS results are typically evaluated visually one at a time. This approach works well only with a small number of files. With many files automated help is needed. Manhattan Plot peaks are difficult to model based on simple mathematical function because they reflect genomic structure and underlying biological aspects in addition to certain expected peak geometry. We tackled this issue by creating a) MH which uses GQS and other parameters to mimic the opinion of an experienced researcher when picking out genome regions with features calling for further attention, b) Cropper that accompanies MH whenever there is a need to study the detected regions up-close. We demonstrate that our tools are fully adequate both in terms of accuracy and speed.

We have developed MH so that it is certain to detect all peaks and it is up to the user to draw the line between the interesting and uninteresting. The MH algorithms can automatically scale to adapt to various peak identities. The peaks are not initially identified based on *p*-value but rather on a collection of peak qualities via specialized data pre-processing and vector fragmentation techniques. Data points with very low *p*-values are ignored if they are not found to belong to high certainty peaks. MH outperforms simple GWAS output screening based on the magnitude of p-value: the low p-value points are combined into regions and the regions, not the individual low p-values, are reported as peaks. This converts the findings into a manageable set that can be further ranked according to the user needs.

MH and Cropper are meant to integrate into the GWAS result analysis workflow. It starts with generation of GWAS summary statistics with GWAS software. All resulting files are then analyzed by MH in the batch mode. The regions of interest are ranked by filtering and sorting the MH output. Only a small number of hits are brought to the researcher’s attention. The researcher can next focus on the short list by using Cropper. The utility of Cropper was assessed by asking the UT scientists using it. The feedback was fully positive ensuring that the tool was needed.

MH is not dealing with issues of biological significance. The peaks are evaluated based solely on the visually observable characteristics. This was the goal because this is also how the scientist evaluates the peaks. The same phenotype can result in Manhattan Plots of different visual qualities depending on the number of subjects and other study characteristics.

MH is not devoid of limitations. It is not currently using the MAF or imputation quality info and thus relies on using quality pre-filtered GWAS files. Also it is not attempting to validate the peaks using non-mathematical methods such as performing database searches or comparing against known genome region associations to find gene identities. Although MH is not providing graphical output this functionality is performed by Cropper. Our future plans include updating the tools based on user feedback.

## Conclusions

We created a system for quickly detecting the interesting genomic regions from GWAS output files. To the best of our knowledge there are no other tools for helping to extend the human eye to a large number of GWAS outputs the same way as MH/Cropper. However these tools are needed. The aim of MH is to do a fast initial screening of data not manageable for the human eye. MH and Cropper together constitute a system that allows the user to qualitatively study a large number of GWAS results.

### Availability and requirements

Project name: Manhattan Harvester.

Project home page: www.geenivaramu.ee/en/tools

Operating system(s): Cross platform.

Programming language: C++/Qt.

Other requirements: Qt4.3 or higher (free from www.qt.io).

License: GNU GPL.

## Additional files

Additional file 1: Supplementary table and figures that show the parameters computed by MH and the Cropper interface. (PDF 679 kb)

Additional file 2: R files showing the computer code used for modeling. (ZIP 2 kb)

## Abbreviations

GHz: Gigahertz
GQS: General Quality Score
GUI: Graphical User Interface
GWAS: Genome-Wide Association Study
KHE(s): Knowledgeable Human Evaluator(s)
MAF: Minor Allele Frequency
MAGNETIC: Metabolites And GeNETIcs Consortium
MB: Megabite
MDS: Method Development Set (subset of the MAGNETIC data set)
MH: Manhattan Harvester
MVS: Method Validation Set (subset of the MAGNETIC data set)
NMR: Nuclear Magnetic Resonance
