# Supplementary material for: Manhattan Harvester and Cropper: a system for GWAS peak detection
Source: BMC Bioinformatics. 2019 Jan 11;20:22. doi: 10.1186/s12859-019-2600-4 (PMC6330393; doi:10.1186/s12859-019-2600-4)

Additional file 1

Table 1: Peak parameters computed by Manhattan Harvester (MH)

MH automatically computes the several parameters for each peak and reports them in the summary files. The following columns appear in the MH output file:

**filename**	name of the input file
**chrom**	chromosome number/name
**range**	range of the peak (in absolute position units)
**max**	maximal –logP value of the peak detected
**bestslope0**	highest absolute slope value of the peak (position vs. -logP) computed using 5-point sliding window
**bestslope**	same as above but each slope value is multiplied by its corresponding –logP value
**monot**	peak monotonity (in fractional units) computed relative to the assumption that –logP values should be monotonously increasing from start to peak and mononononously decreasing from peak to end
**balance**	peak balance; number of points to the left of the max(-logP) divided by the number of points to the right of the max(-logP); non-linear with respect to peak quality
**multip**	peak multiplicity; indicates what fraction of points have –logP=max(-logP)
**reps**	peak repetitions; indicates what fraction of points have a –logP equal to that of their neighbor
**ratio**	height to width ratio calculated as $–logP*10^6/width$
**kolmo**	Kolmogorov-Smirnoff test of normality; 0 = normality condition not satisfied, 1= normality condition satisfied; not suitable for comparing peaks of very different size
**range**	width of the peak in bp
**count**	number of points in the peak
**spacing**	mean distance between points
**balance**	number of all points to the right of max(-logP) divided by the number of points to the left of max(-logP); non-linear with respect to peak quality
**skew**	all –logP values to the right of max(-logP) divided by all –logP values to the left of max(-logP); non-linear with respect to peak quality
**vbal1**	number of data points with –logP values >= mean(-logP) divided by number of data points with –logP values < mean(-logP)
**vbal2**	same as above but instead of point counts the cumulative –logP values are used
**maxbyMean**	max(-logP) divided by mean(-logP)
**GQS**	General Quality Score of the peak (1-5)

Fig. 1. The two views of Cropper: global view opens first to show all chromosomes, local view shows just one chromosome.

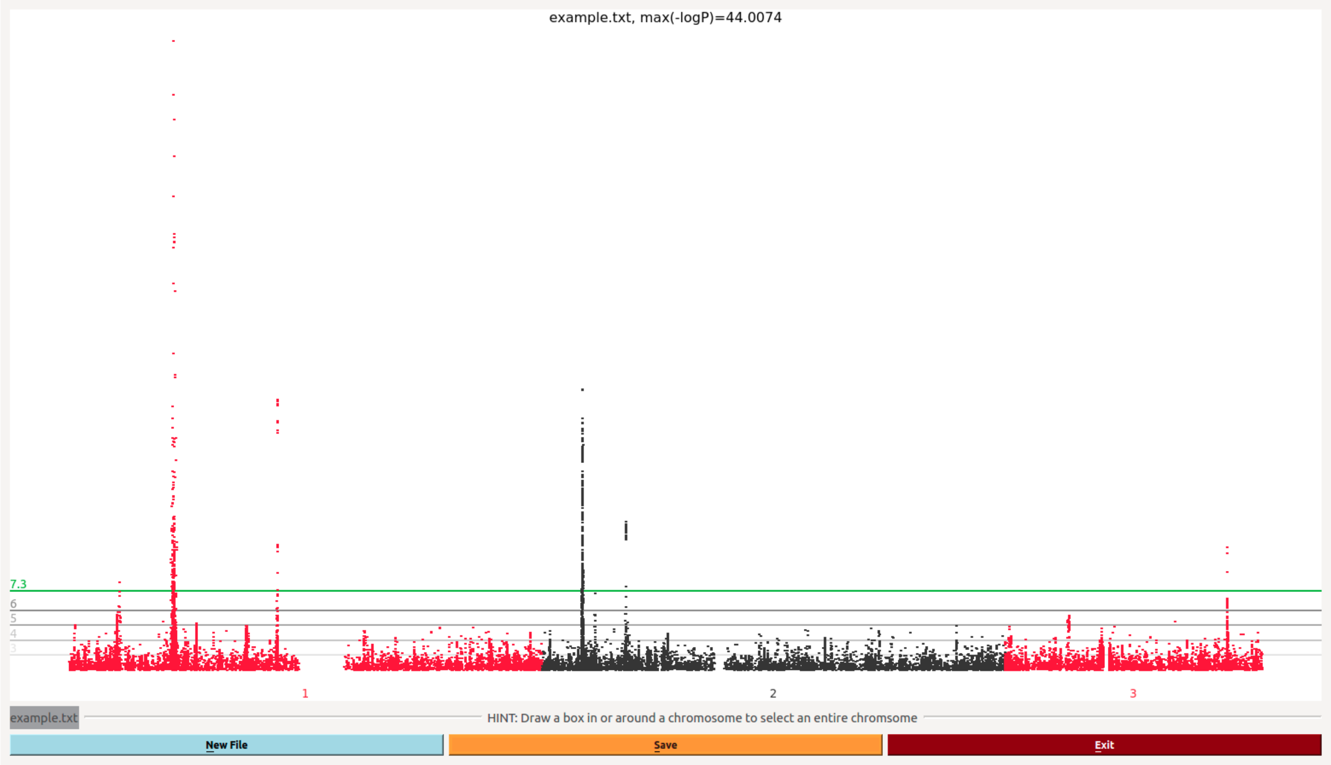

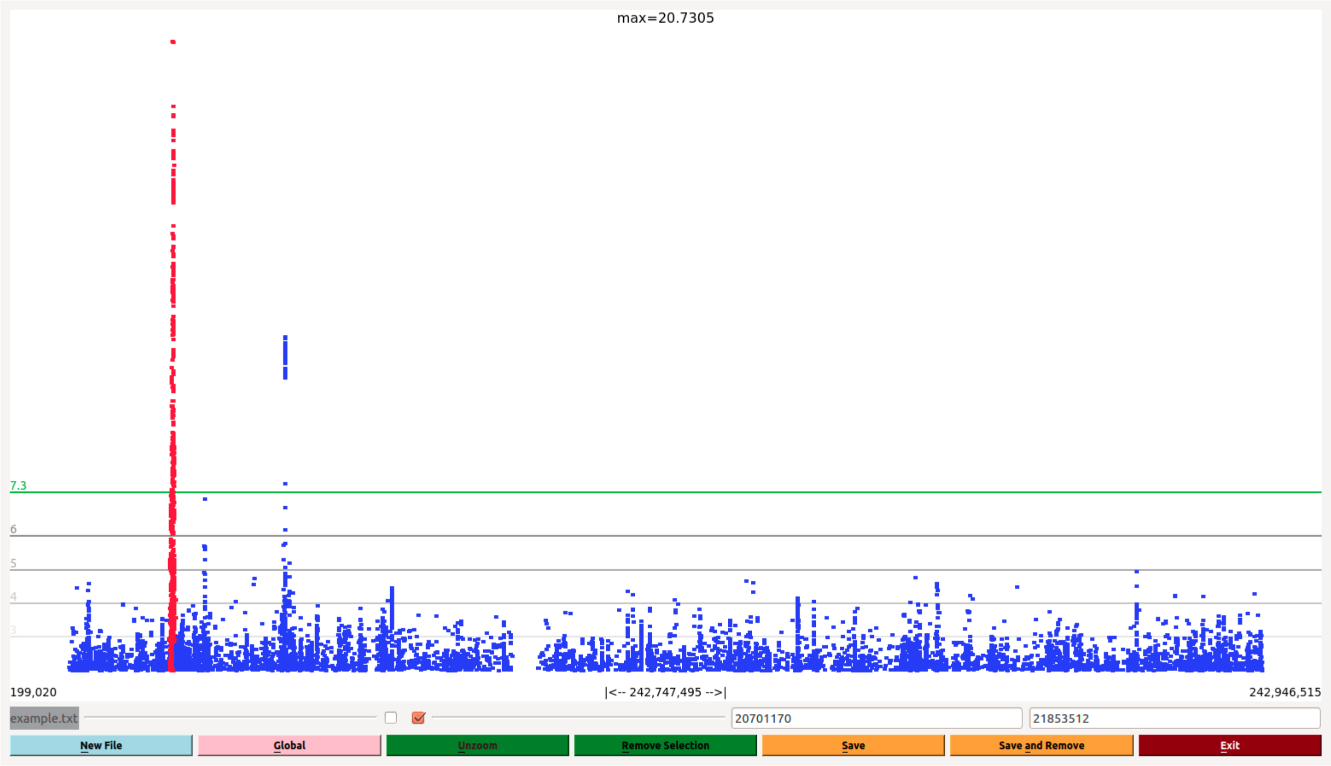

Fig.2. Correlations between the MH output parameters used as attributes in GQS modeling.

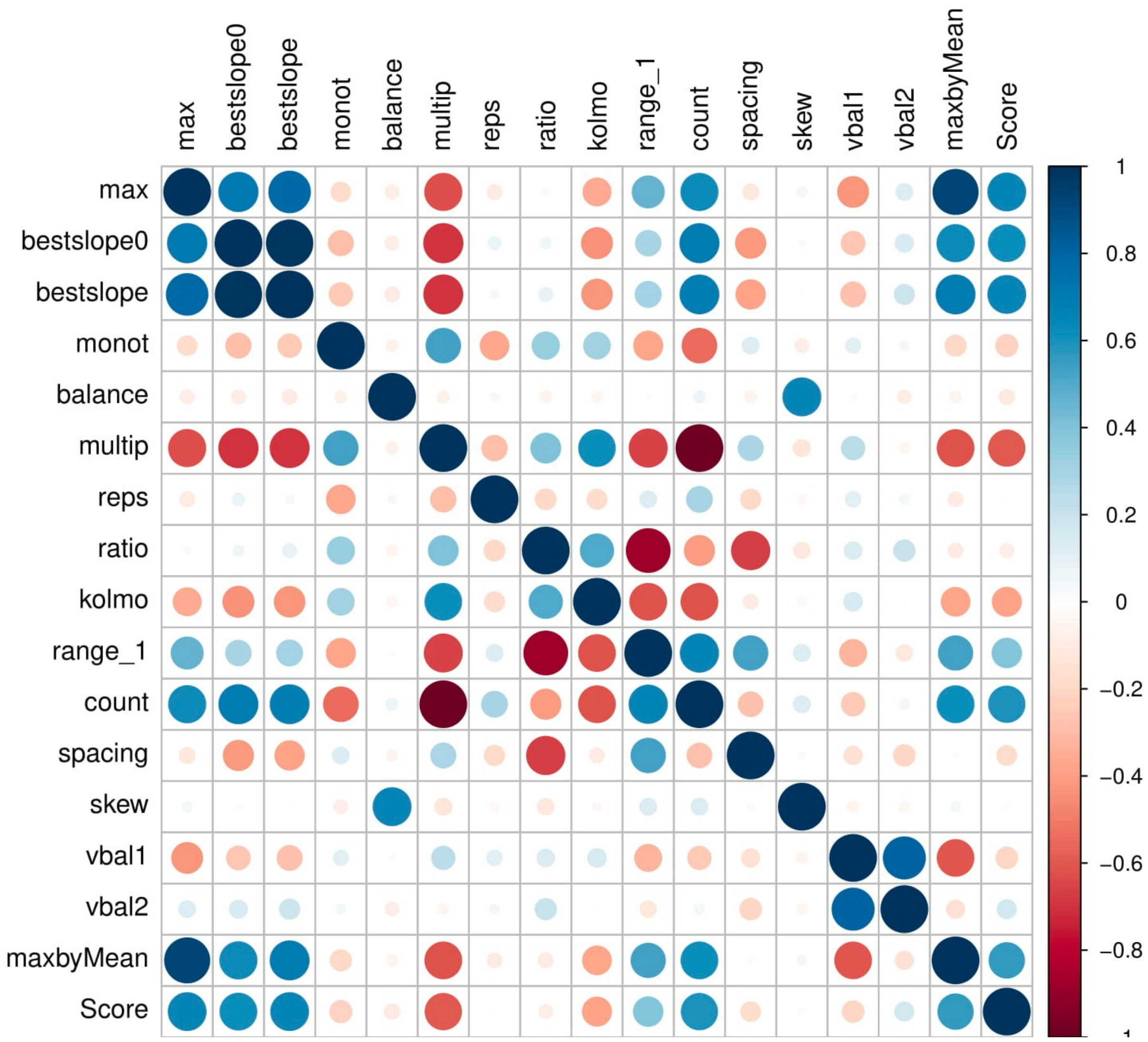

Fig. 3. Correlations of MH output parameters with the score variable grouped by KHEs.

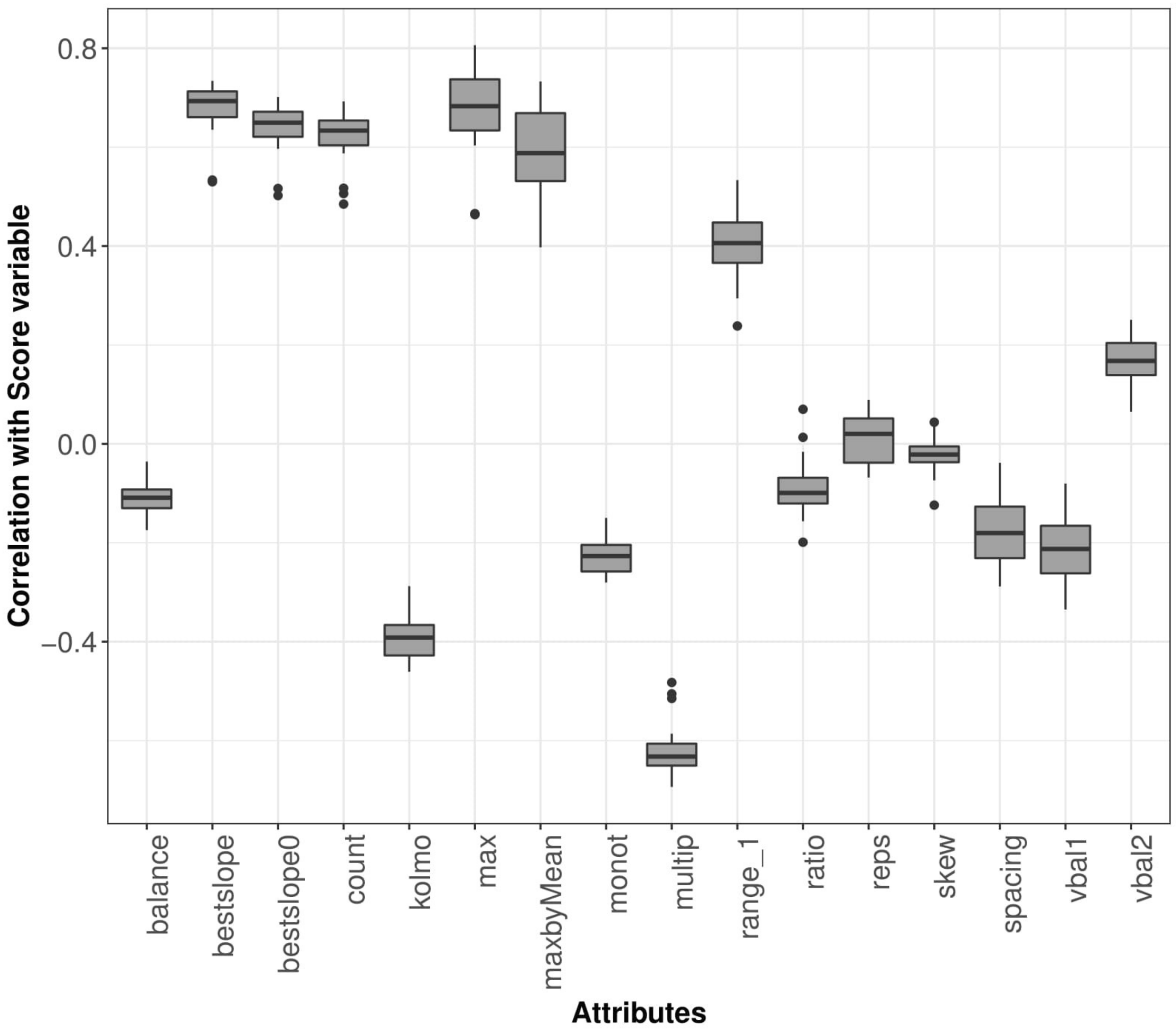

Fig. 4. Examples of peaks (exactly as shown to KHEs) with average scores by KHEs and the GQS assignment by Manhattan Harvester. The green line corresponds to P = 5 * 10^{-8}, gray lines correspond to P = 10^{-6}, 10^{-5}, $10^{-4,}$ 10^{-3} with lower P-values displaying darker color.

KHEs = 4.95; GQS = 5

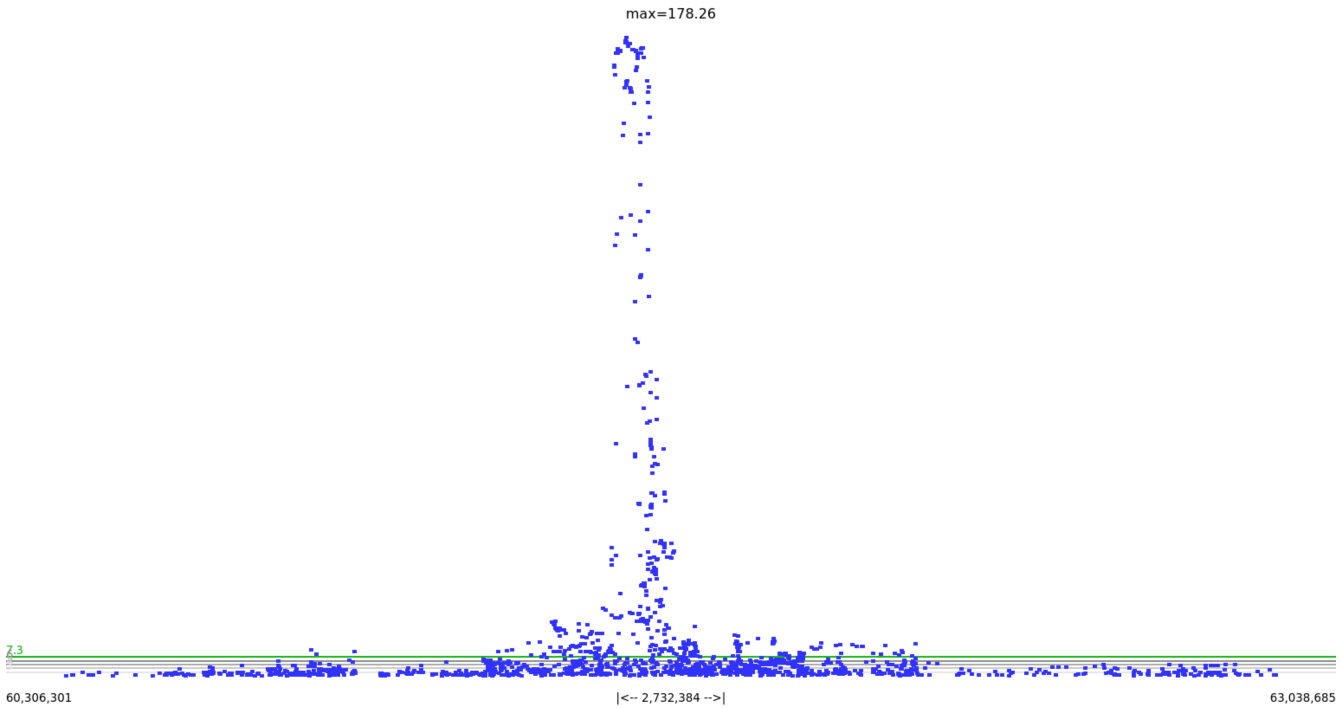

KHEs = 5; GQS = 4.99

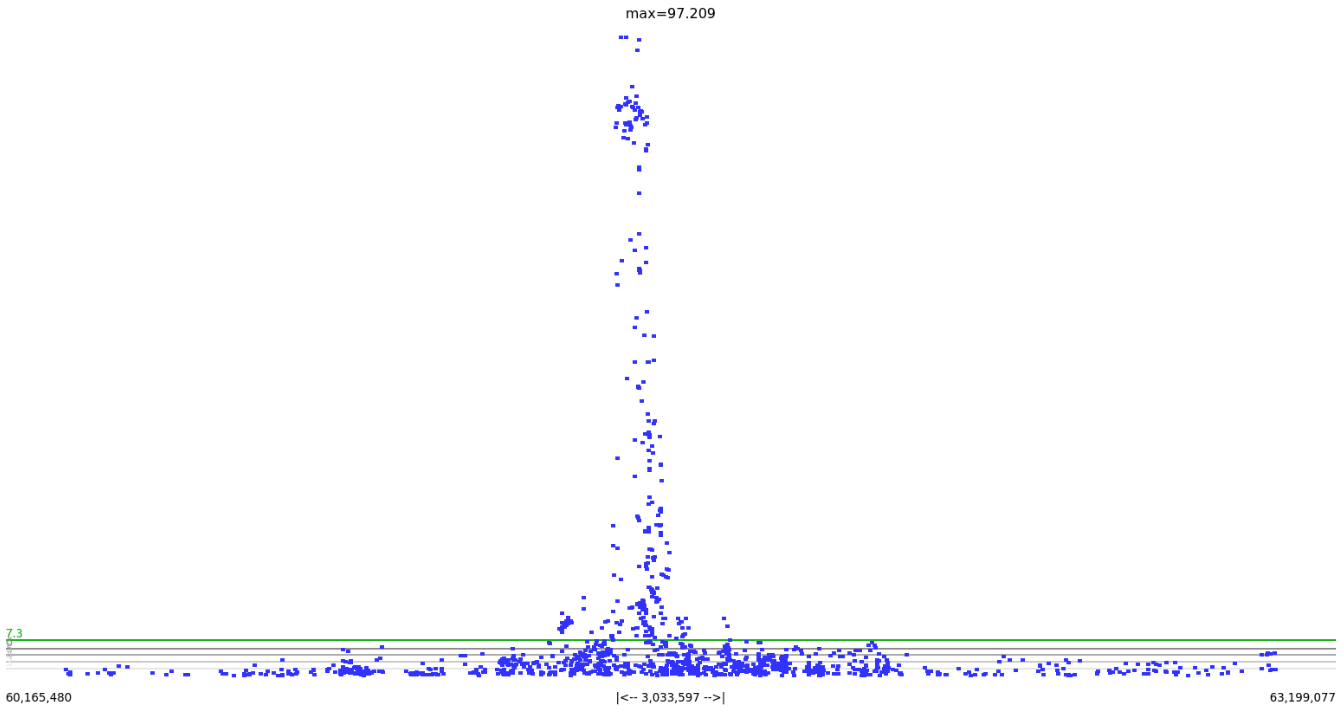

KHEs = 4; GQS = 3.94

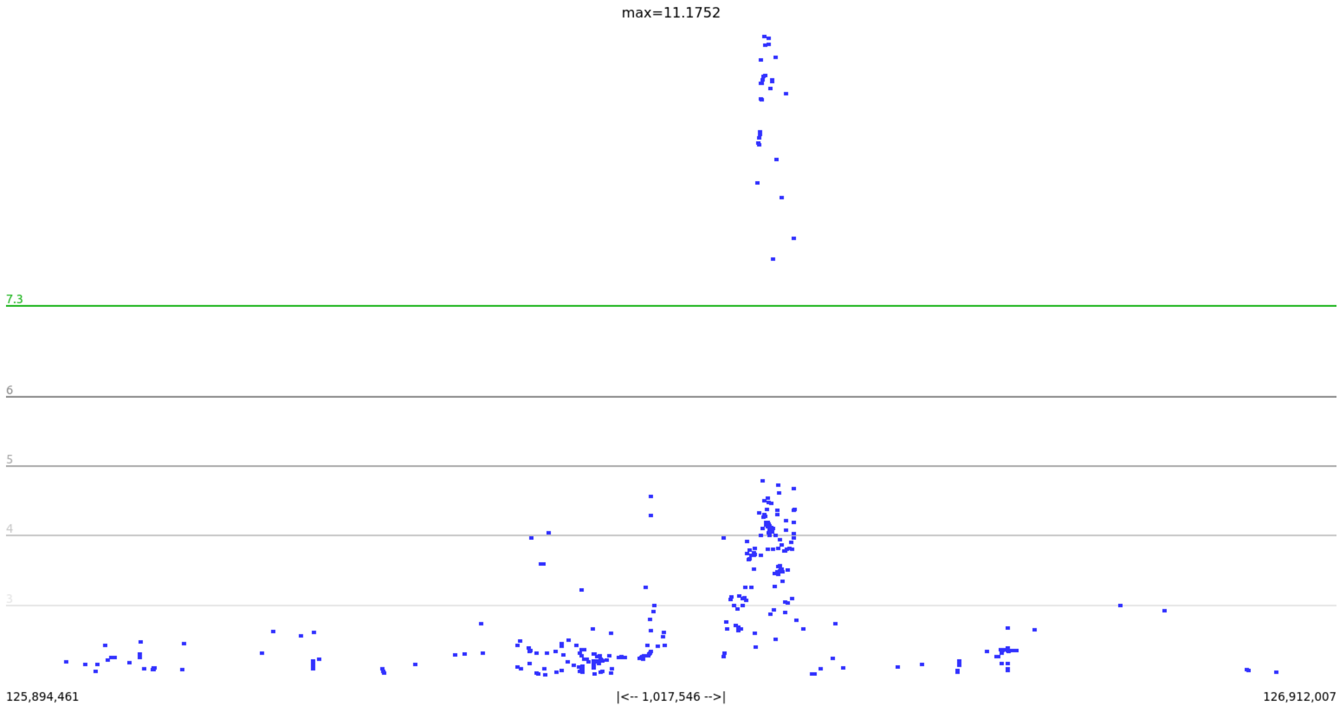

KHEs = 4.1; GQS = 4.29

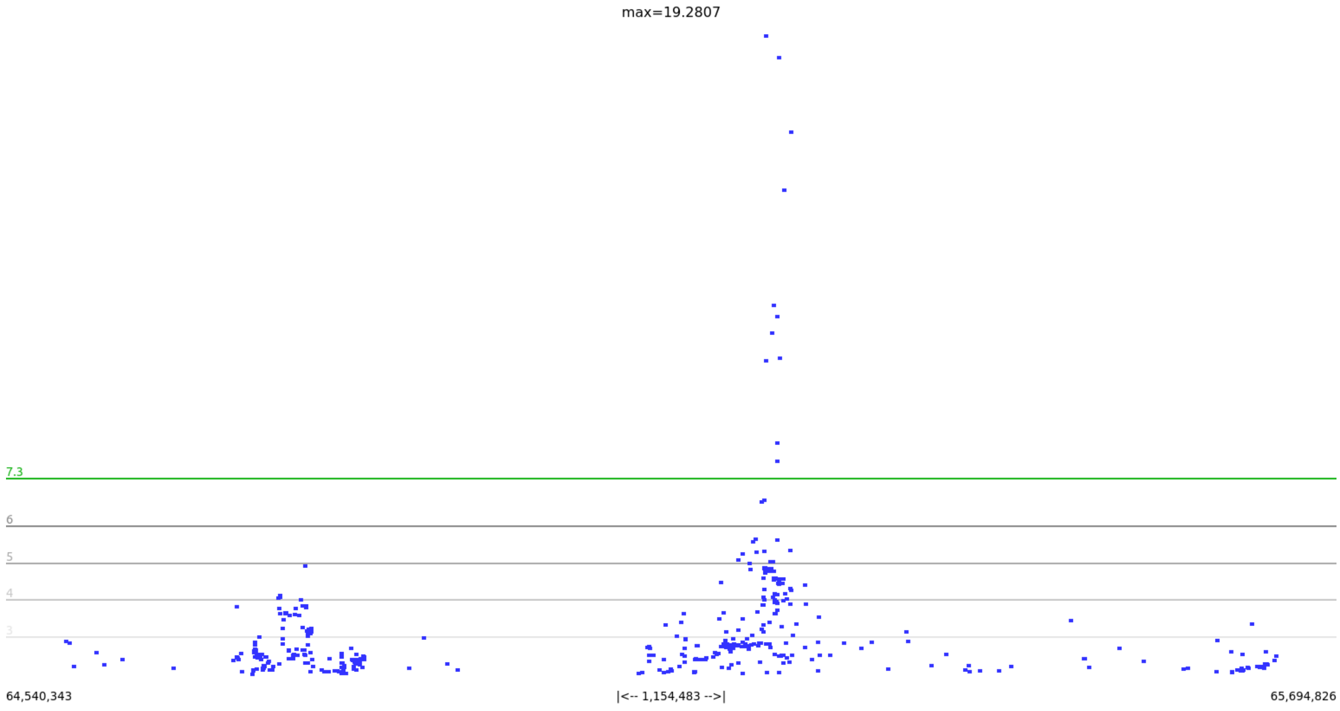

KHEs = 2.95; GQS = 3.12

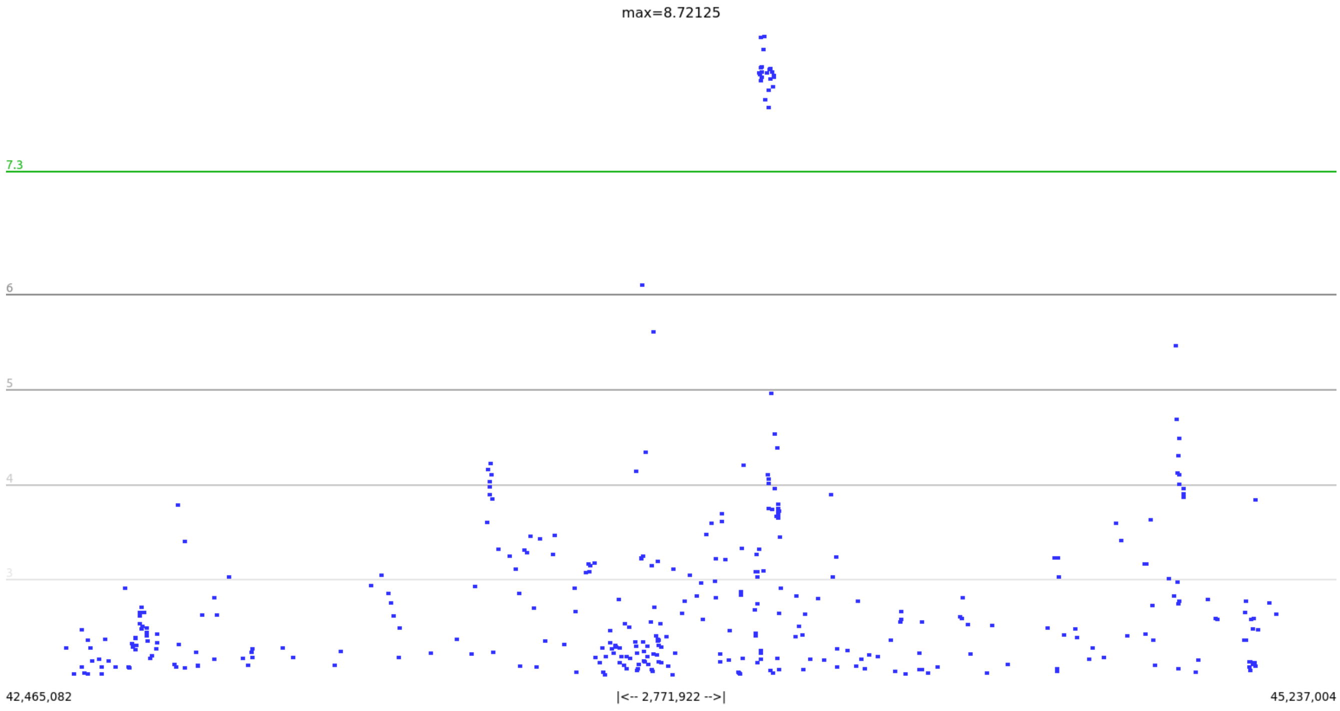

KHEs = 3; GQS = 2.72

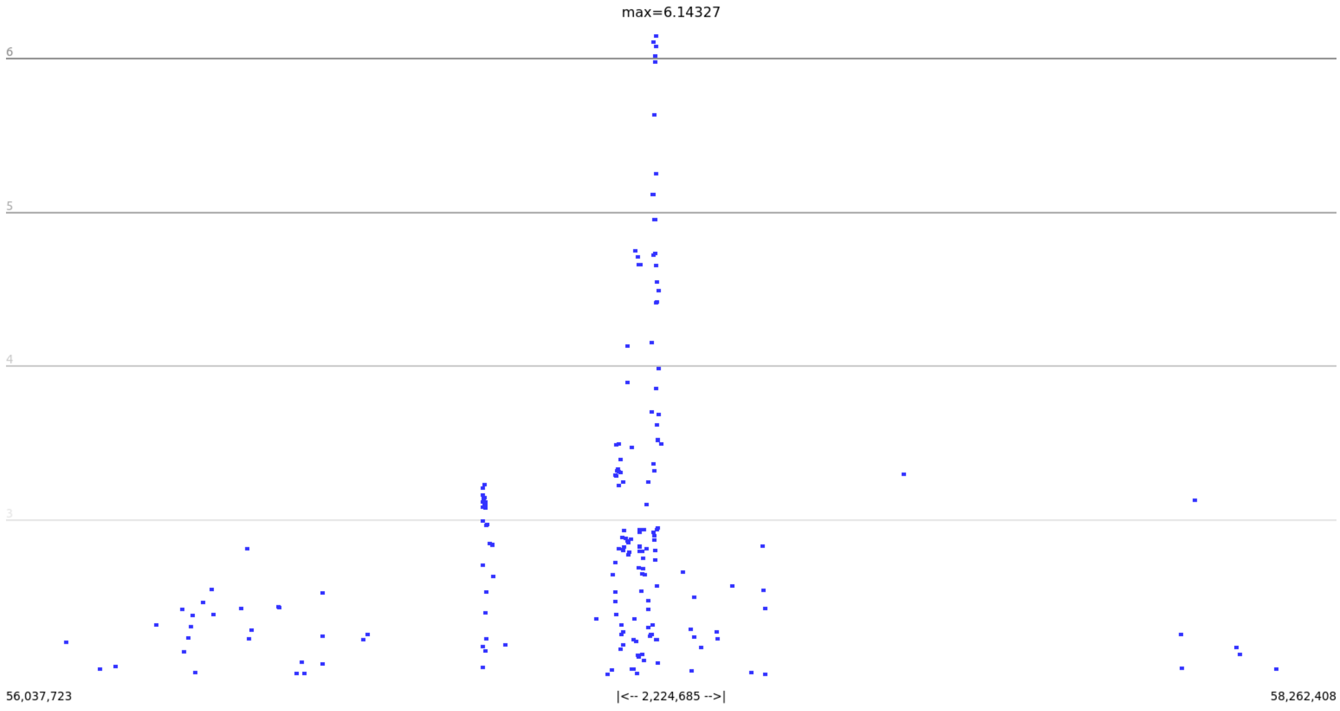

KHEs = 2.1; GQS = 1.76

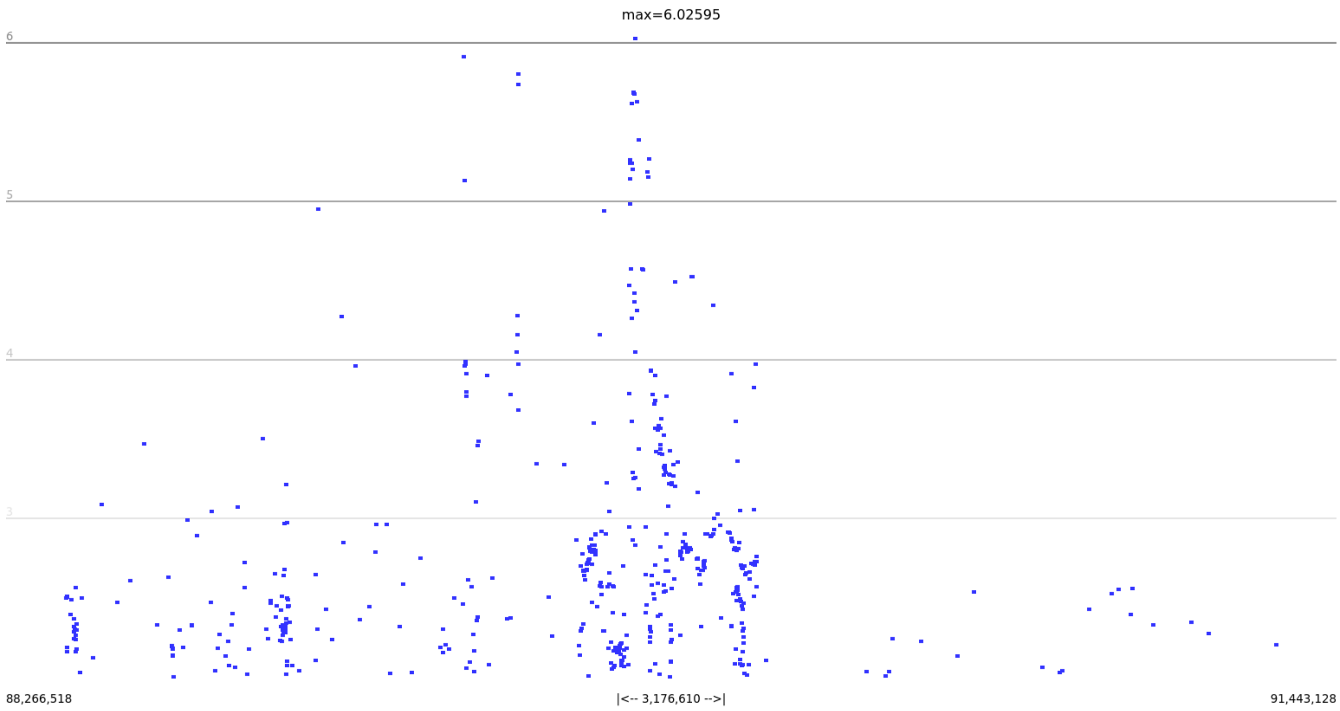

KHEs = 2.1; GQS = 2.98

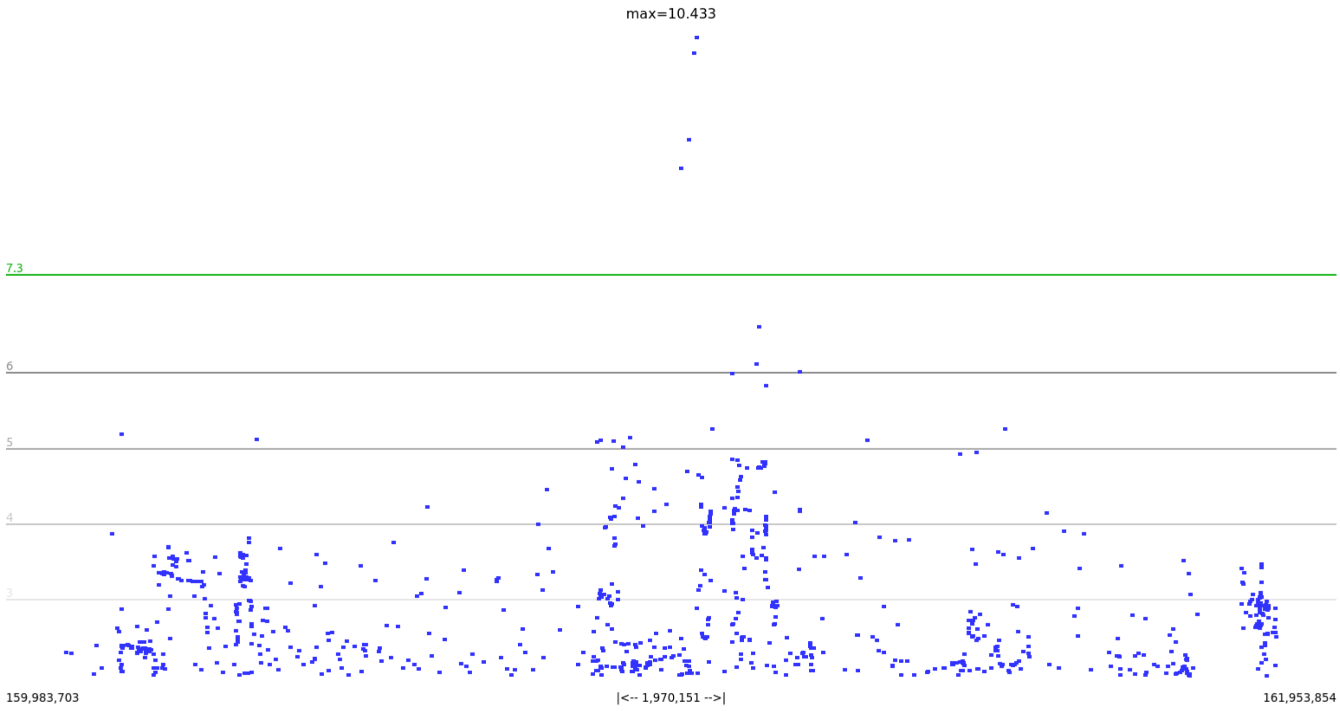

KHEs = 1.1; GQS = 1.77

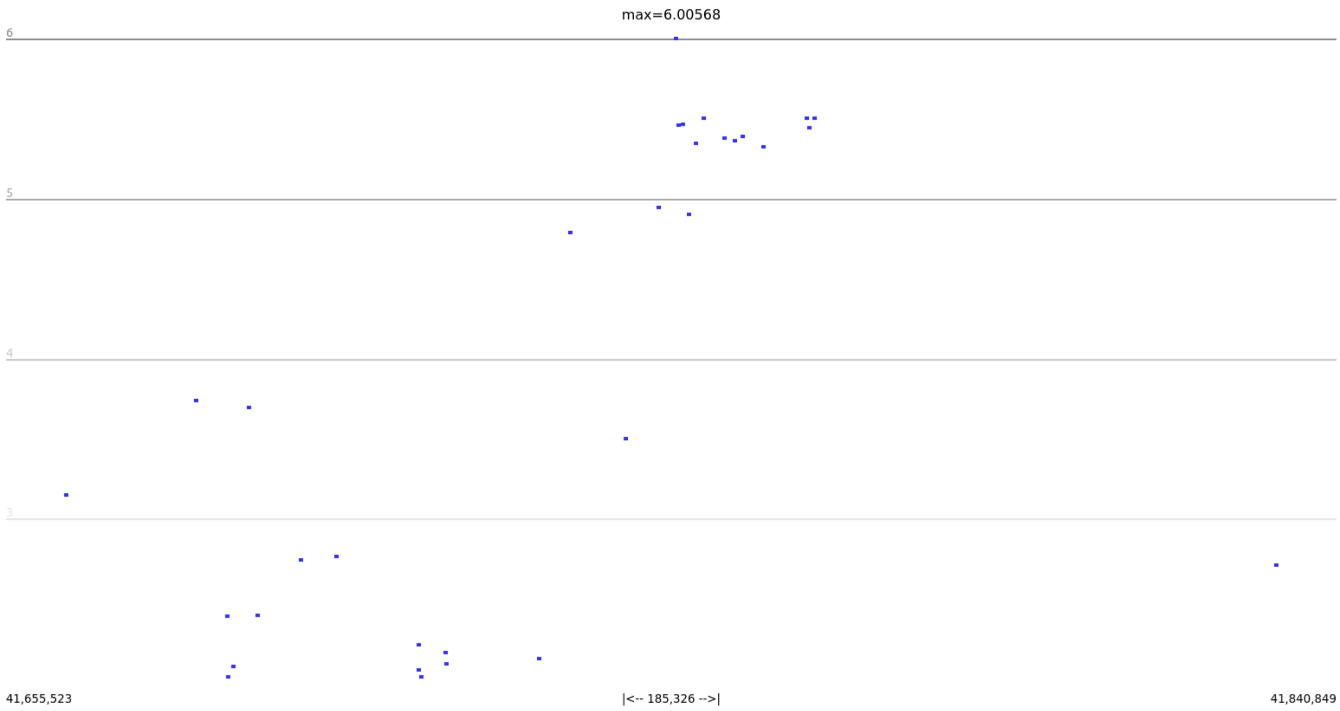

KHEs = 1.15; GQS = 2

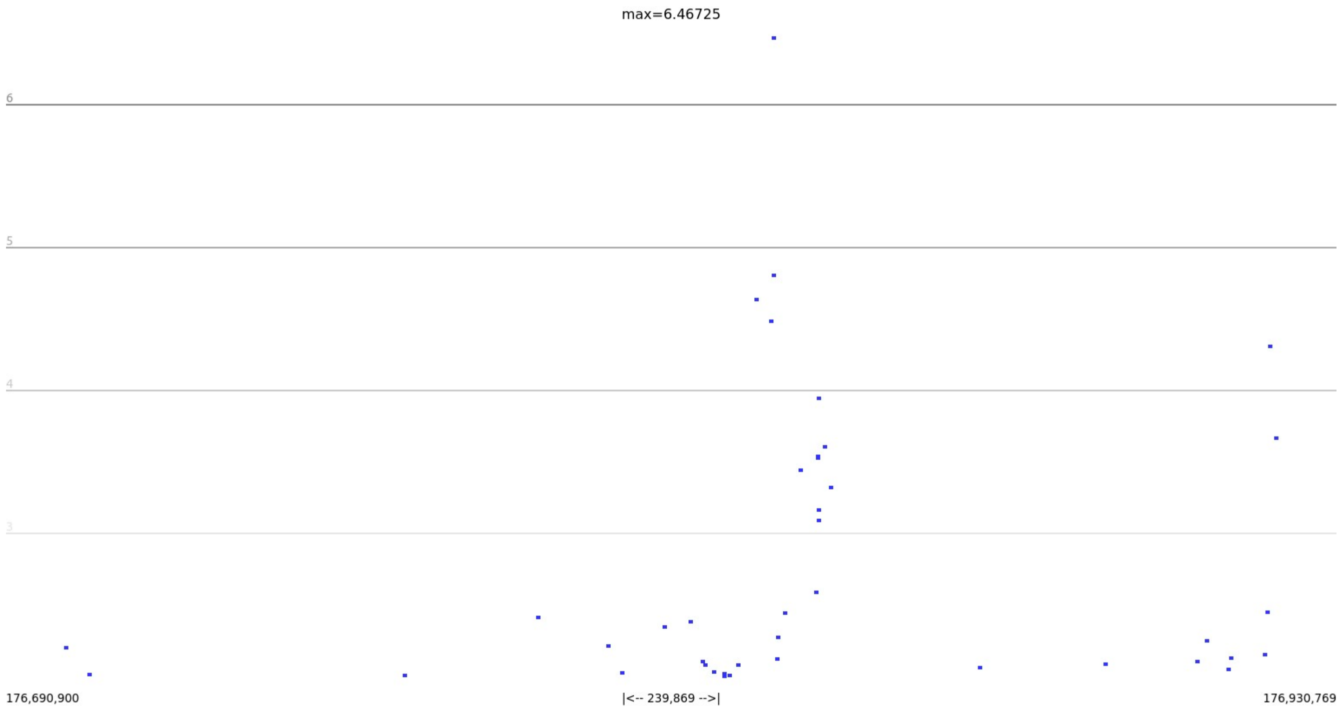

Fig. 5. Manhattan Plot showing the data set used for MH execution speed testing (summarized in Table 1 in the main text).

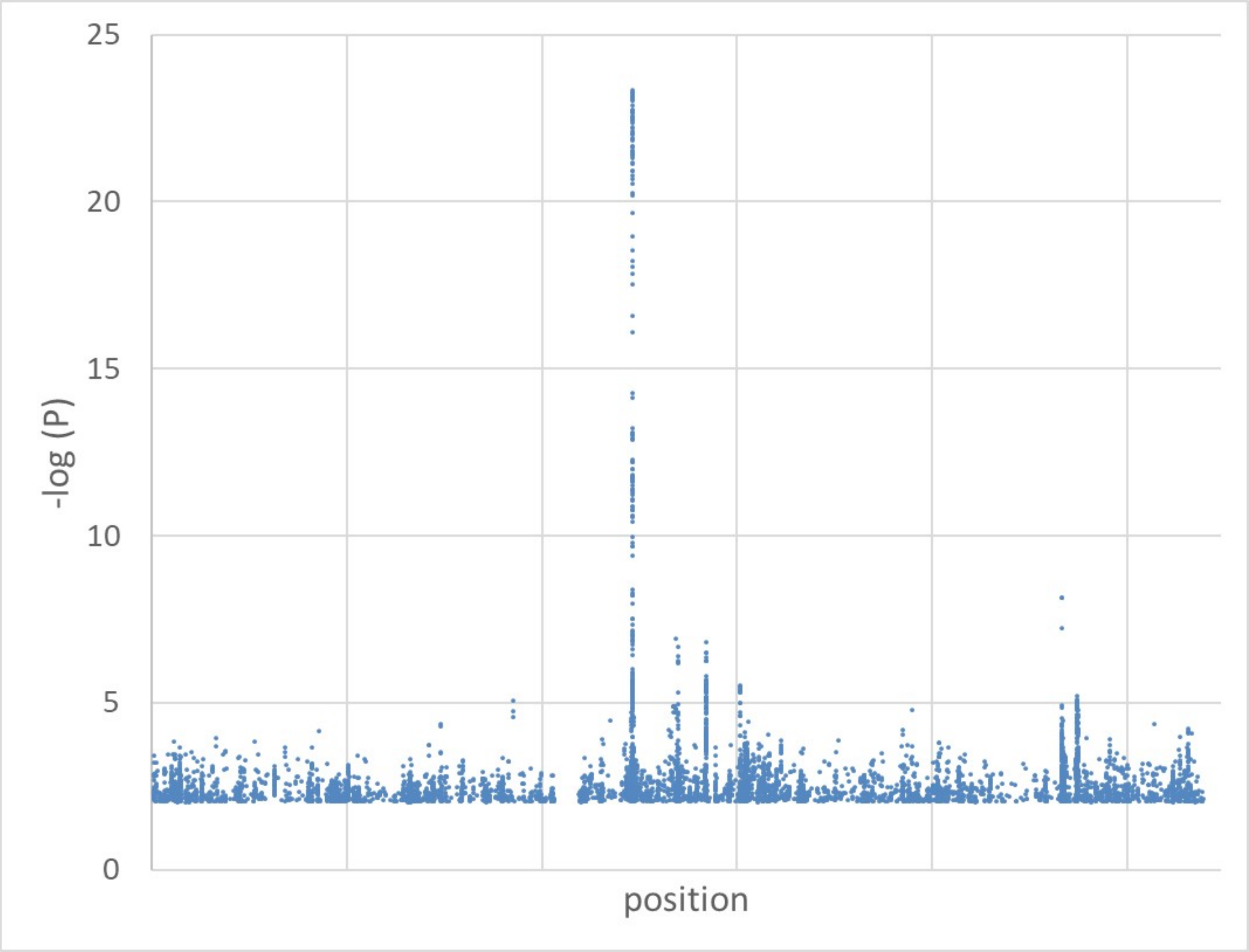

Supplement: Supplementary file 1 — Supplementary table and figures that show the parameters computed by MH and the Cropper interface. (PDF 679 kb) [file 12859_2019_2600_MOESM1_ESM.pdf]
